# Altered Effective Connectivity of Precuneus Subregions and Associated Gene Profiles in Chronic Insomnia Disorder Patients Before and After Treatment

**DOI:** 10.1002/brb3.71448

**Published:** 2026-05-19

**Authors:** Zhiguo Guo, Leyi Zhang, Yiding Han, Haohao Yan, Ping Yao, Dongsheng Lv, Yonggui Yuan, Jingping Zhao, Lixia Chen, Wenbin Guo

**Affiliations:** ^1^ Inner Mongolia Autonomous Region Mental Health Center Hohhot Inner Mongolia Autonomous Region China; ^2^ Department of Psychiatry, National Clinical Research Center for Mental Disorders, and National Center for Mental Disorders The Second Xiangya Hospital of Central South University Changsha Hunan China; ^3^ Department of Psychosomatics and Psychiatry, Zhongda Hospital, School of Medicine, Jiangsu Provincial Key Laboratory of Brain Science and Medicine Southeast University Nanjing China

**Keywords:** estazolam, Granger causality analysis, hyperarousal model, modified suanzaoren decoction, pharmacology

## Abstract

**Background:**

Previous studies have highlighted the crucial role of the precuneus in the hyperarousal state of insomnia. However, the causal effects between its subregions and other regions remain unclear. Both traditional Chinese medicines (TCMs) and benzodiazepines are effective in insomnia treatment, but limited studies have compared the therapeutic mechanisms of these two treatments.

**Methods:**

This study included 82 chronic insomnia disorder (CID) patients and 80 healthy controls (HCs). CID patients were assigned to receive either modified suanzaoren decoction, a TCM for treating insomnia, or estazolam treatment for 6 weeks. Granger causality analysis seeded on the precuneus subregions was employed to detect effective connectivity (EC) alterations at pre‐ and post‐treatment. Moreover, a neuroimaging–transcription association analysis was conducted.

**Results:**

Compared to HCs, CID patients showed increased EC from the precuneus to the right postcentral/precentral gyrus (PoCG/PrCG) and left lingual gyrus. Meanwhile, decreased ECs from the bilateral PoCG/PrCG, right cerebellum crus VIIB, and right calcarine cortex to the precuneus were observed. After 6 weeks of treatment, significant group‐by‐time interactions on ECs from the bilateral midcingulate cortex to precuneus, as well as from the bilateral midcingulate cortex, left PrCG, and right supramarginal gyrus to precuneus were observed. Genes associated with these ECs were identified.

**Conclusions:**

The bidirectional ECs between the precuneus and regions in the sensory‐motor and visual networks in CID may reflect both hyperarousal states and compensatory responses to counteract abnormal hyperarousal. TCM tended to alleviate the hyperarousal state in CID while benzodiazepines induced an adaptive regulation due to their rapid sedative effects.

**Trial Registration:**

Clinical trial registration number: NCT06452953

## Graphical abstract

Granger causality analysis seeded on the precuneus subregions was employed to detect effective connectivity alterations at pre‐ and post‐treatment. The treatment included 6 weeks of modified suanzaoren decoction (a TCM widely used to improve insomnia) or estazolam. Finally, correlation analyses and a neuroimaging–transcription association analysis were conducted.

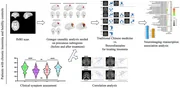

## 1 Introduction

Chronic insomnia disorder (CID) is characterized by difficulty initiating sleep at bedtime, early‐morning awakening, difficulty returning to sleep, and complaints of poor sleep quality and short overall sleep duration. Notably, the potential pathophysiological mechanism underlying CID remains unclear, with the most prevailing being the hyperarousal model (Riemann et al. 2010). This model hypothesizes that heightened sensory and cognitive processing results in increased vulnerability to environmental or other stimuli, which eventually interferes with sleep initiation and maintenance (Riemann et al. 2010).

According to the hyperarousal model, a lack of deactivation of the default mode network (DMN) might serve as a functional basis for CID (Marques et al. 2018; Zhao et al. 2020). The DMN is a task‐negative network involved in self‐referential processes, such as introspection and self‐awareness (Andrews‐Hanna et al. 2014). CID patients often present overconcerns about their sleep complaints as well as explicit intention and effort to fall asleep, which might activate the DMN (Marques et al. 2015; Riemann et al. 2010). As a hub of the DMN, most previous studies identified hyperactivity in the precuneus in insomnia patients relative to healthy controls (HCs; Aquino et al. 2024; Zhao et al. 2020). Studies examining abnormal functional connectivity (FC) of the precuneus have yielded various results. There was increased FC between the precuneus and pallidum, supraoccipital gyrus, and postcentral gyrus (Lee et al. 2018; Xia et al. 2022), while decreased FC was observed between the precuneus and nucleus accumbens, cingulate gyrus, right angular gyrus, middle frontal gyrus, and left inferior temporal gyrus in insomnia patients relative to HCs (Li et al. 2023; Xia et al. 2022; Zhang et al. 2024). These divergent results might be attributed to the distinct functions of different subregions of precuneus. Moreover, the precuneus is engaged in various functions, such as self‐generated thought, awareness, visuospatial imagery, and episodic memory retrieval (Andrews‐Hanna et al. 2014; Cavanna and Trimble 2006; Dörfel et al. 2009; Vogt and Laureys 2005; Wenderoth et al. 2005). These various roles of the precuneus further underscore the existence of its subregions. Previous studies have identified distinct roles for the precuneus subregions, with the anterior, central, and posterior precuneus involved in sensorimotor, cognitive/associative, and visual functions, respectively (Margulies et al. 2009). Zhu et al. identified selective functional impairments of the precuneus subregions in major depressive disorder, underscoring the importance of precuneus divisions (Zhu et al. 2018). Notably, given the prevalent impairment of the precuneus in CID, exploring the functional changes in precuneus subregions might provide further insight into its specific role in CID.

However, most previous studies have focused on the activation and connectivity of the precuneus, omitting the directed influence between the precuneus and other regions. Granger causality analysis (GCA) provides a novel perspective to better understand the neural mechanisms underlying mental disorders (Leroy et al. 2022; Yu et al. 2024). Compared to FC analysis, which reflected connectivity between brain areas that share functional properties, GCA can identify brain regions that may serve as sources or targets of directed influence for a selected region of interest (ROI), elucidating causal interactions between regions. Specifically, if the past time series of X and Y predict the current time series of Y more accurately than the past Y time series alone, this indicates a directed influence from X to Y. Using GCA, we could explore the causal relationship between precuneus subregions and other regions across the whole brain in CID patients.

Currently, benzodiazepines (BDZs) are widely prescribed for treating CID. They potentiate the inhibitory neurotransmitter gamma‐aminobutyric acid (GABA) by binding to the BDZ‐type receptor on the GABA ion channel. BDZs primarily target the spinal cord (muscle relaxation), brainstem and cerebellum (ataxia), and limbic and cortical regions (emotional and behavioral processes; Lader 2011). However, they are often associated with dependence and withdrawal symptoms. Traditional Chinese medicine (TCM) has emerged as an alternative therapy with fewer dependency issues compared to Western medications (Shi et al. 2016; Yeung et al. 2012). Suanzaoren decoction (SZRD) is a widely used TCM for treating insomnia, with its efficacy validated in randomized controlled trials (Xie et al. 2013; Zhou et al. 2018) and rodent studies (Dong et al. 2021). Limited mechanistic research on SZRD suggested that it might improve sleep by regulating neurotransmitters (e.g., activating serotonin, increasing GABA receptors, reducing Orexin‐A, antagonizing glutamic acid) and reducing inflammation (Dong et al. 2021; Shi et al. 2016; Wang et al. 2023; Yang et al. 2012). In clinical practice, modified SZRD (MSZRD) is customized to different insomnia patterns according to TCM theory by adding one or more herbs to the original SZRD formula (Song et al. 2020). While TCMs and BDZs appeared to exert distinct therapeutic mechanisms for improving insomnia, few neuroimaging studies have investigated their neural therapeutic mechanisms. Existing studies have reported that TCMs and BDZs could induce functional or structural alterations in insomnia patients, suggesting underlying therapeutic targets (Chen et al. 2022; Feng et al. 2019). Methodological heterogeneity across studies limited the comparisons between these two treatment approaches. In the current study, estazolam and MSZRD were selected as treatment options for CID patients.

This study was designed to investigate whole‐brain directed or effective connectivity (EC) of the precuneus subregions and to compare the neural therapeutic mechanisms of estazolam and MSZRD. We also integrated these neuroimaging phenotypes with gene expression profiles in the Allen Human Brain Atlas (AHBA) database to identify CID‐related genes. Based on the hyperarousal model and the various functions of the precuneus, we hypothesized that there would be increased ECs from the precuneus to other regions involved in sensory, motor, visual, and cognitive regions, indicating physiological and cognitive hyperarousal. Due to the chronic duration of insomnia, ECs from these regions to the precuneus might decrease as a form of negative feedback regulation in the brain. Genes associated with these EC alterations would be identified. After 6 weeks of treatment, estazolam and MSZRD would induce different EC alterations, indicating their distinct therapeutic mechanisms.

## 2 Methods

### 2.1 Participants

Eighty‐two CID patients (16 males, 66 females) were recruited from the Center of Mental Health, Inner Mongolia Autonomous Region. CID patients were included if they met the following criteria: (1) primary insomnia; (2) independently diagnosed by two proficient psychiatrists according to the International Classification of Sleep Disorders third edition (ICSD‐3); (3) aged 18–65 years; (4) at least 9 years of formal education; and (5) right‐handed.

Only patients with yin‐deficiency and fire‐excess syndrome, as diagnosed by a Chinese medicine practitioner, were eligible for this study. Yin‐deficiency and fire‐excess syndrome is a common pattern of insomnia according to TCM (Poon et al. 2012). This syndrome manifests as insomnia along with symptoms such as headaches, irritability, night sweats, dry mouth, palpitations, red tongue, and rapid pulse. According to TCM treatment, Succinum (Hupo, HP) could alleviate yin‐deficiency and fire‐excess syndrome by calming the mind, clearing internal heat, and restoring emotional balance. HP was added to our SZRD formula to enhance its sleep‐treatment effects.

Exclusion criteria for patients were as follows: (1) heavy tea drinkers, cigarette smokers, and coffee‐dependent individuals; (2) night‐shift workers; (3) severe physical diseases, other mental disorders, or severe obstructive sleep apnea; (4) pregnancy or breastfeeding; and (5) contraindications to MRI scanning.

Eighty HCs (18 males, 62 females), matched by age, sex, and education level, were recruited from the community. These individuals were excluded from having any personal or family history of psychiatric disorders. All HCs reported experiencing restorative and satisfactory sleep, maintaining regular sleep habits, and did not report significant stress levels.

The study was approved by the Medical Ethics Committee of Inner Mongolia Autonomous Region Mental Health Center. All subjects provided written informed consent before participation.

### 2.2 Study Design

After enrollment, CID patients completed 2 weeks of washout period and underwent a brain MRI scan and clinical assessment. Patients were then assigned to either the MSZRD group or the estazolam group based on their treatment preferences. MSZRD was composed of *Ziziphus jujuba* Mill. (Suanzaoren, SZR) 30.0 g, *Ligusticum acuminatum* Franch. (Chuanxiong, CX) 15.0 g, *Anemarrhena asphodeloides* Bunge. (Zhimu, ZM) 15.0 g, *Poria cocos* (Schw.) Wolf. (Fuling, FL) 15.0 g, *Glycyrrhiza uralensis* Fisch. (Gancao, GC) 3.0 g, and HP 3.0 g. The SZRD formula granules and HP powder were sourced from Sichuan New Green Pharmaceutical Technology Development Co. Ltd., Batch No. 2107020. Patients in the MSZRD group were instructed to mix SZRD formula granules and decoct them into a solution. Patients were then instructed to place HP powder in their mouths and consume it with the prepared SZRD decoction half an hour after breakfast and dinner. Estazolam tablets were sourced from Shandong Xinyi Pharmaceutical Co. Ltd., Batch No. H370230047, Strength 1 mg/tablet. Patients in the estazolam group took estazolam alone (1 mg/day, before bedtime). All patients received sleep‐related education and were required to avoid other sedative‐hypnotic medications during the treatment. After 6 weeks of treatment, all patients underwent an MRI scan and clinical assessment again. HCs underwent a single MRI scan and clinical assessment once at baseline.

### 2.3 Assessment

The Insomnia Severity Index (ISI), Pittsburgh Sleep Quality Index (PSQI), and polysomnography (PSG) were used to assess sleep quality. The ISI is a self‐report measure for quantifying insomnia severity. It comprises seven items that assess difficulties in falling asleep, staying asleep, and waking up too early, satisfaction with current sleep pattern, interference with daily functioning, and noticeability and concern about current sleep problems (Bastien et al. 2001). The PSQI assessed individual sleep quality and disturbances over the past month. It is a seven‐item inventory designed to assess subjective sleep quality, sleep latency, duration, efficiency, and disturbances, use of sleep medications, and daytime dysfunction (Buysse et al. 1989). PSG provides objective sleep assessments, such as measurements of sleep latency and total sleep time. It uses various measures (e.g., electroencephalogram, electrooculogram, electromyogram, and electrocardiogram) to discriminate sleep stages and monitor physiological activity across different organ systems (Jafari and Mohsenin 2010). PSG is a gold‐standard measurement of sleep, as it provides information that is used to rule out other sleep disorders, such as sleep apnea, that might contribute to insomnia (Morin et al. 2015).

In addition, the depression and anxiety symptoms of all subjects were measured using the Hamilton Depression Scale (HAMD) and Hamilton Anxiety Scale (HAMA).

### 2.4 Imaging Data Acquisition and Preprocessing

Resting‐state fMRI data were collected using a Siemens Skyra 3.0 T scanner with the following parameters: repetition time/echo time = 2000 ms/30 ms, 37 slices, 64 × 64 matrices, flip angle 90°, 24 cm FOV, 4 mm slice thickness, no gap, and 240 volumes (480s). Participants were asked to remain still, close their eyes, and stay awake.

The imaging data were preprocessed in MATLAB R2018 b (http: //www. mathworks.com) with the SPM12 and RESTplus software (Jia et al. 2019). The procedures comprised the following steps: deletion of the first 10 images, slice timing, motion correction, normalization to the standardized Montreal Neurological Institute (MNI) space with the voxel size of 3 mm × 3 mm × 3 mm, smoothing (Gaussian kernel with a full width at half maximum [FWHM] of 6 mm), linear trend removal, and regression of the Friston‐24 head motion parameters as well as signals from cerebrospinal fluid and white matter centered region. The global signal was not removed. Subsequently, band‐pass filtering (0.01–0.08 Hz) was performed. Subjects who exceed a maximum of 2 mm displacement in the *x*‐, *y*‐, or *z*‐axis and 2° angular motion in any direction would be excluded from the final analysis.

### 2.5 ROI Selection and GCA

**FIGURE 1:**
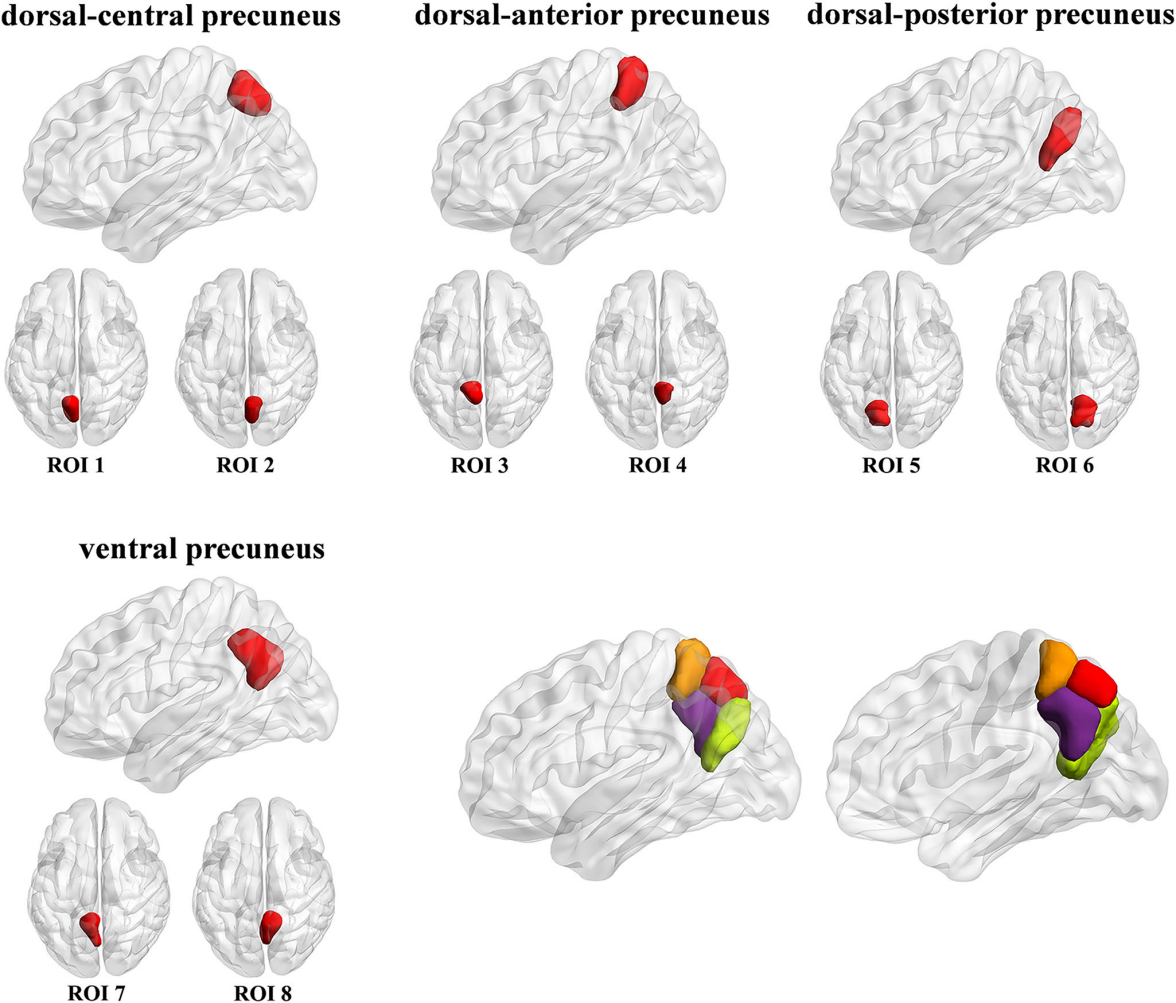
Granger causality analysis seeded on the precuneus subregions in chronic insomnia patients. L, left; R, right; ROI, regional of interest; ROI 1, dorsal–central precuneus, medial area 7 (L); ROI 2, dorsal–central precuneus, medial area 7 (R); ROI 3, dorsal–anterior precuneus, medial area 5 (L); ROI 4, dorsal–anterior precuneus, medial area 5 (R); ROI 5, dorsal–posterior precuneus, dorsomedial parietooccipital sulcus (L); ROI 6, dorsal–posterior precuneus, dorsomedial parietooccipital sulcus (R); ROI 7, ventral precuneus, area 31 (L); ROI 8, ventral precuneus, area 31 (R).

Based on a recent connectivity‐based parcellation study (Fan et al. 2016), the precuneus was parcellated into eight subregions (ROI1‐8) according to their specific anatomical and FC patterns (Figure 1 and Table S1). EC was calculated using GCA in the RESTplus toolbox (Jia et al. 2019). For detailed information on GCA, refer to the Supporting Information.

### 2.6 Statistical Analysis

Comparison of demographic and clinical data across two patient groups and HCs, as well as between two treatment groups were compared using the Kruskal–Wallis tests, chi‐square test, two‐sample *t*‐tests, or Mann–Whitney *U*‐tests in SPSS 25.0 software, if applicable. A mixed‐effects model was used to evaluate the effects of group (estazolam or MSZRD), treatment time, and group‐by‐time interaction on sleep quality.

Two‐sample *t*‐tests were conducted on the Granger causality maps to identify the case–control difference and the difference between the two treatment groups at baseline, with gender, age, education level, and head motion as covariates. The Gaussian random field (GRF) method was used for multiple comparison correction with *p* < 0.001 at the voxel level and *p* < 0.05 at the cluster level. A mixed‐model ANOVA based on the DPABI toolbox was performed to detect neuroimaging changes in pre‐ and post‐treatment between treatment groups, with gender, age, education level, and head motion as covariates (Chao‐Gan and Yu‐Feng 2010). Post hoc tests were performed to further examine specific group differences. Bonferroni correction was used for multiple comparisons. To further examine whether individual characteristics might modulate the treatment effects between MSZRD and estazolam, we included potential moderating factors such as age, sex, and baseline disease severity as higher‐level variables in the mixed‐effects model. Specifically, interaction terms between these variables, treatment group (MSZRD vs. estazolam), and time (pre‐ vs. post‐treatment) were tested to examine whether any specific subgroup exhibited differential treatment effects.

Correlation analyses were employed to assess the association between neuroimaging anomalies and clinical variables (i.e., PSQI, ISI, HAMA, and HAMD scores) based on the DPABI toolbox, with age, sex, education levels, and head movement as covariates. The GRF method was used for multiple comparisons.

### 2.7 Neuroimaging–Transcription Association Analysis

The gene expression data were sourced from the publicly available AHBA database (Hawrylycz et al. 2012). Using the abagen toolbox (version 0.1.1; https://github.com/rmarkello/abagen), an expression matrix of 15,633 × 116 (genes × regions) was obtained. Cross‐sample correlations between case–control EC alterations and gene expression data were performed by Spearman correlation to identify genes whose expression levels were correlated with spatial differences in CID patients. For each ROI‐based EC analysis, both the *X‐to‐Y* and *Y‐to‐X* directions were analyzed separately. Bonferroni correction was used for multiple comparisons with a corrected *p* value of 0.05/15633. The effects of autocorrelation in neuroimaging data can lead to inflated *p* values in transcriptome‐neuroimaging correlation analyses. The BrainSMASH toolbox addresses this by generating 1000 surrogate brain maps that preserve the original autocorrelation properties while representing randomized phenotypes (Burt et al. 2020). Spearman correlation analysis was repeated between the surrogate maps and the gene expression data. Genes whose expression levels correlated with 50 or more of the 1000 surrogate brain maps were excluded from the analysis. Subsequently, we conducted Gene Ontology (GO) and Kyoto Encyclopedia of Genes and Genomes (KEGG) term analysis with these genes related to case–control EC alterations using Metascape (https://metascape.org/).

## 3 Results

### 3.1 Demographic and Clinical Characteristics

A total of 82 CID patients and 80 HCs were included in our analysis. Of these, 15 patients failed to finish the follow‐up, and one was excluded due to excessive head movement. Finally, 41 patients in the MSZRD group and 25 patients in the estazolam group were included for a longitudinal analysis. The age, gender, and education level across the two treatment groups and HCs were comparable (*p* > 0.05). Between the two treatment groups, there was no significant difference in terms of their scores of HAMA, HAMD, PSQI, ISI, and sleep indicators measured by PSG (*p* > 0.05). Details were presented in Table S2.

In patient groups, the mean duration of MSZRD and estazolam treatment was 50.37 (SD = 16.18) and 46.64 (SD = 16.93) days. The clinical characteristics of CID patients who finished the follow‐up were displayed in Table S3. Using the mixed‐effects model, there were significant time, group, and group‐by‐time effects on the total scores of PSQI (*p* < 0.001) and the scores of sleep duration in PSQI (*p* < 0.001). Detailed information was shown in Figure S1 and Table S4.

### 3.2 GCA and Correlation Analysis

**FIGURE 2:**
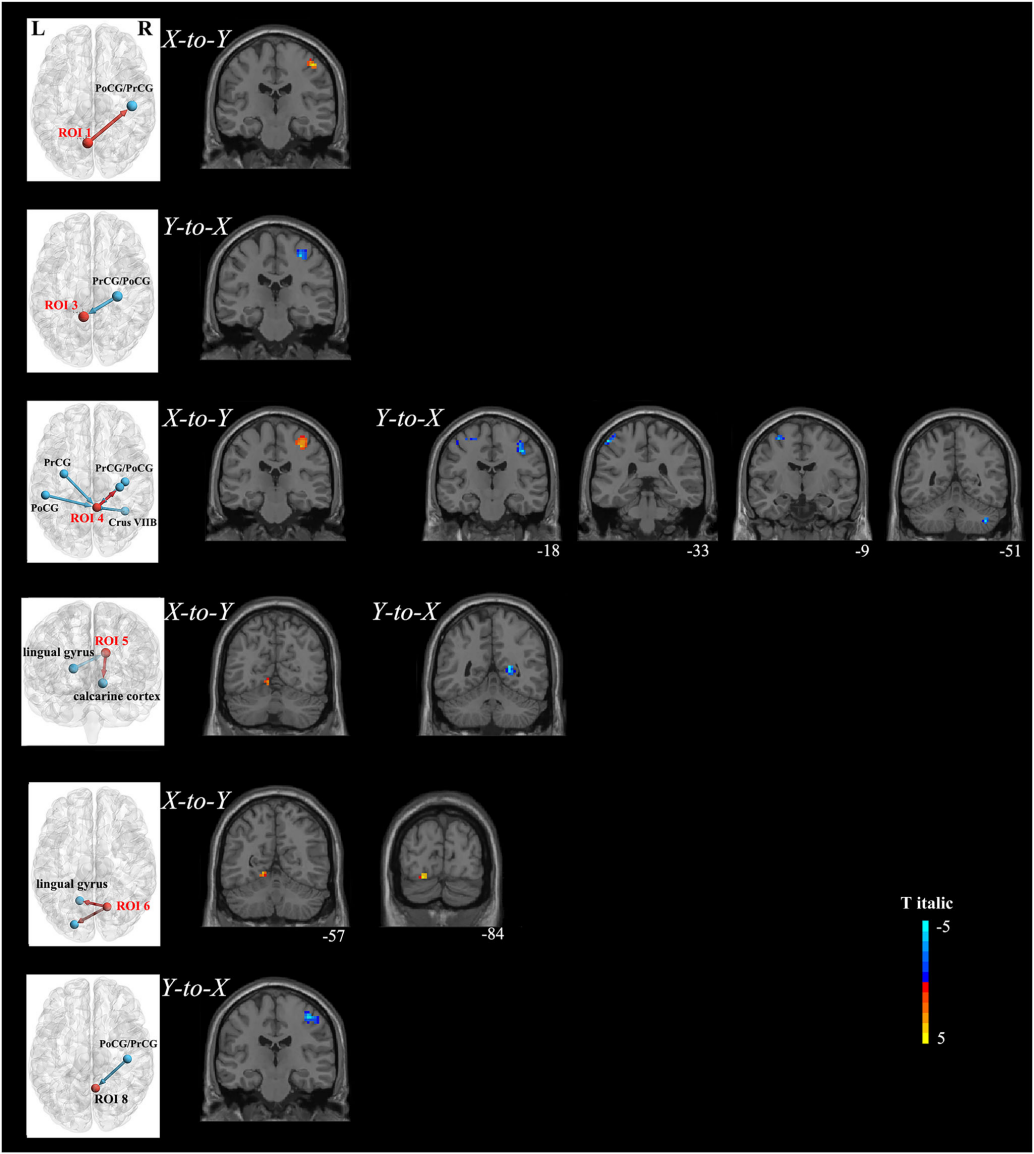
Abnormal effective connectivity in patients with chronic insomnia disorder at baseline compared to healthy controls. The arrows indicate the direction of effective connectivity. X‐to‐Y indicates connectivity from the precuneus (ROI) to other brain regions, while Y‐to‐X indicates connectivity from other brain regions to the precuneus (ROI). Red arrows represent increased connectivity, while blue arrows represent decreased connectivity. The color bar displays the *T* values corresponding to the strength of the connectivity differences. L, left; R, right; ROI, regional of interest; ROI 1, dorsal–central precuneus, medial area 7 (L); ROI 3, dorsal–anterior precuneus, medial area 5 (L); ROI 4, dorsal–anterior precuneus, medial area 5 (R); ROI 5, dorsal–posterior precuneus, dorsomedial parietooccipital sulcus (L); ROI 6, dorsal–posterior precuneus, dorsomedial parietooccipital sulcus (R); ROI 8, ventral precuneus, area 31 (R).

**FIGURE 3:**
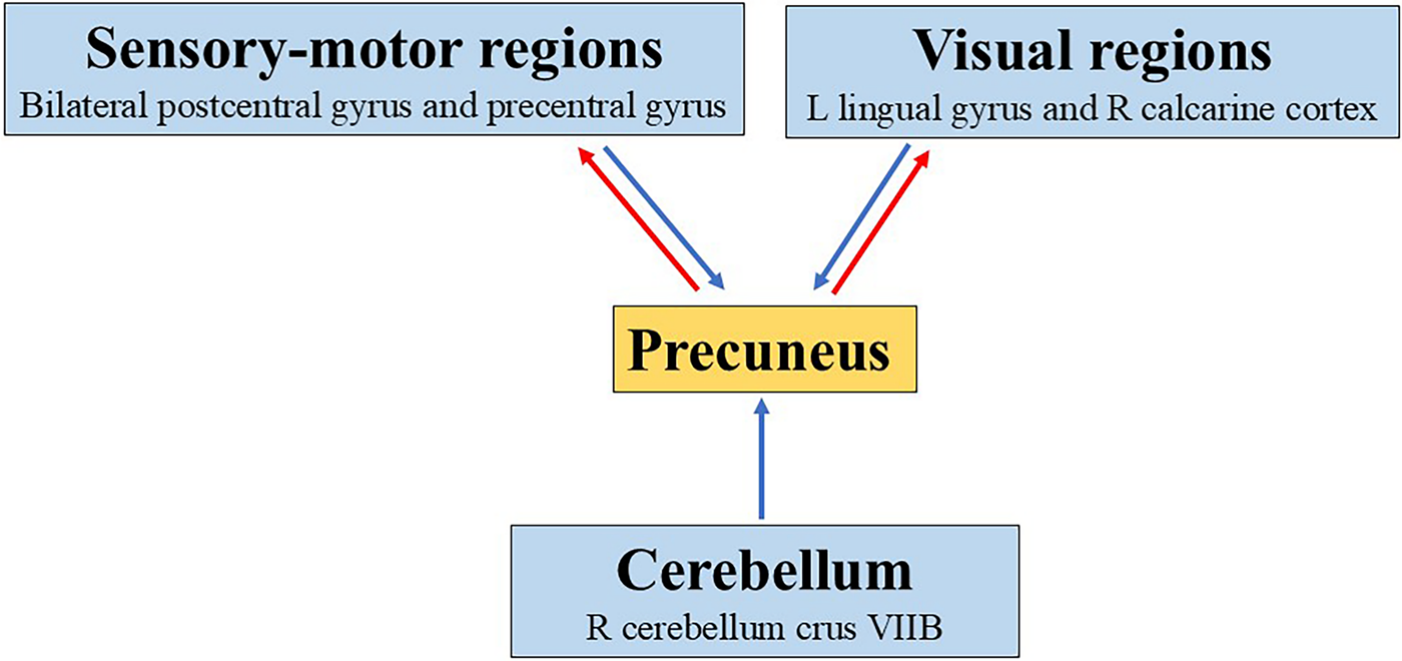
Schematic illustration of effective connectivity between the precuneus subregions and other regions in patients with chronic insomnia disorder at baseline compared to healthy controls. Colored lines represent direction of causal influence: red lines indicate increased effective connectivity and blue lines indicate decreased effective connectivity. L, left; R, right.

At baseline, CID patients exhibited increased ECs from the precuneus to right postcentral/precentral gyrus (PoCG/PrCG) and left lingual gyrus (LG) compared to HCs. In whole‐brain‐to‐precuneus GCA, there were decreased ECs from the bilateral PoCG/PrCG, right cerebellum crus VIIB, and right calcarine cortex to precuneus (Figures 2 and 3 and Table 1). When comparing baseline EC differences between the two groups, patients in the MSZRD group showed decreased EC from the left Cerebellum Crus II to the precuneus compared with those in the estazolam group (Table S5). To further evaluate the potential impact of baseline EC differences between treatment groups, we performed an additional voxel‐wise two‐sample *t*‐test on individual change maps (ΔEC = post − pre) between the MSZRD and estazolam groups. No significant group differences were observed after GRF correction.

**TABLE 1:** The differences in effective connectivity between patients and healthy controls.

ROIs	Brain regions	MNI (*x*, *y*, *z*)	*T* value	Cluster size
ROI 1 (left dorsal–central precuneus, medial area 7)				
*X‐to‐Y*	R postcentral/precentral gyrus	45, −21, 54	4.45	31
ROI 3 (left dorsal–anterior precuneus, medial area 5)				
*Y‐to‐X*	R precentral/postcentral gyrus	30, −24, 54	−4.43	49
ROI 4 (right dorsal–anterior precuneus, medial area 5)				
*X‐to‐Y*	R postcentral/precentral gyrus	33, −24, 51	4.60	93
*Y‐to‐X*	L precentral/postcentral gyrus	39, −18, 45	−4.60	68
	L postcentral gyrus	−51, −33, 57	−4.48	34
	L precentral gyrus	−30, −9, 60	−4.27	29
	R cerebellum crus VIIB	39, −51, −45	−4.46	25
ROI5 (left dorsomedial parietooccipital sulcus of precuneus)				
*X‐to‐Y*	L lingual gyrus	−9, −63, −6	4.05	36
*Y‐to‐X*	R calcarine cortex	21, −51, 9	−4.42	26
ROI 6(right dorsomedial parietooccipital sulcus of precuneus)				
*X‐to‐Y*	L lingual gyrus	−15, −57, −6	4.19	55
	L lingual gyrus	−21, −84, −15	4.33	29
ROI 8 (right ventral precuneus, area 31)				
*Y‐to‐X*	R postcentral/precentral gyrus	42, −21, 54	−4.91	59

Abbreviations: L, left; MNI, Montreal Neurological Institute; R, right; ROI, regions of interest.

Several significant correlations were identified, including positive correlations between PSQI scores and ECs from the precuneus to the left cerebellum and from the SMG to the precuneus, as well as a negative correlation from the precuneus to the superior medial frontal gyrus. Moreover, there were positive correlations between the ISI scores and the ECs from the right SMG/PoCG and bilateral superior temporal gyrus to precuneus. For HAMD scores, there were negative correlations with the ECs from the left calcarine gyrus/precuneus and superior frontal gyrus to precuneus, as well as a positive correlation with the ECs from the right SMG to precuneus (Figure S2 and Table S6).

**FIGURE 4:**
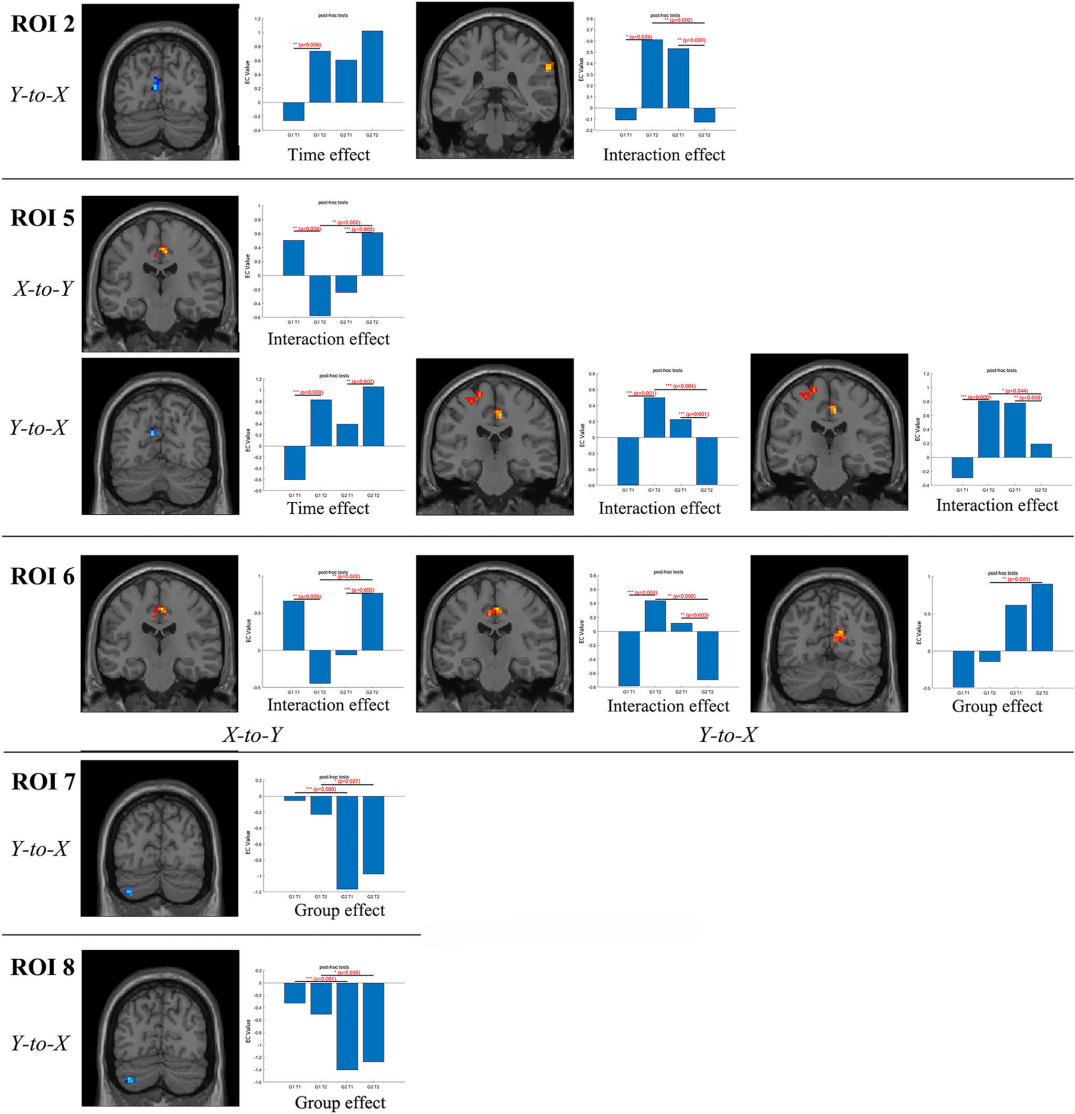
Post hoc *t*‐test results from the mixed effects analysis. All results are based on the effective connectivity abnormalities between the precuneus and the whole brain. X‐to‐Y indicates connectivity from the precuneus (ROI) to other brain regions, while Y‐to‐X indicates connectivity from other brain regions to the precuneus (ROI). Brain slices are used for visualization, and bar charts represent the effective connectivity values of the corresponding brain regions (for both treatment groups at baseline and post‐treatment). Significant *p* values are provided. The specific direction of change for each significant effect can also be found in Table 2. G1, Estazolam group; G2, Suanzaoren decoction group; L, left; R, right; ROI, regional of interest; ROI 2, dorsal–central precuneus, medial area 7 (R); ROI 5, dorsal–posterior precuneus, dorsomedial parietooccipital sulcus (L); ROI 6, dorsal–posterior precuneus, dorsomedial parietooccipital sulcus (R); ROI 7, ventral precuneus, area 31 (L); ROI 8, ventral precuneus, area 31 (R); T1, baseline; T2, follow‐up.

Following treatment, post hoc test results from the mixed‐model ANOVA were displayed in Figure 4 and Table 2. Significant group‐by‐time interaction effects on ECs were observed:(1) from precuneus to bilateral midcingulate cortex (MCC); (2) from bilateral MCC to precuneus; (3) from the right supramarginal gyrus (SMG) to precuneus; (4) from left PrCG to precuneus. Moreover, there was a significant time effect on EC from the left calcarine cortex to precuneus and significant group effects on ECs from the right calcarine cortex and left Cerebellum Crus II to precuneus. When considered moderating effects of sex, age, and baseline ISI severity on these results, no significant effects were found for these factors (Table S7).

**TABLE 2:** Post hoc *t*‐test results from the mixed effects analysis.

ROIs	Mix effects	Brain regions	MNI (*x*, *y*, *z*)	*T*/*F* value	Direction of change	Cluster size
ROI 2 (right dorsal–central precuneus, medial area 7)						
*Y‐to‐X*	Time effect	L calcarine cortex	−6, −75, 12	−4.52	Both groups: EC increased	22
	Interaction effect	R supramarginal gyrus	60, −33, 33	23.15	Estazolam: EC increased MSZRD: EC decreased	26
ROI 5 (left dorsomedial parietooccipital sulcus of precuneus)						
*X‐to‐Y*	Interaction effect	Bilateral midcingulate cortex	3, −21, 48	22.02	Estazolam: EC decreased MSZRD: EC increased	42
*Y‐to‐X*	Time effect	L calcarine cortex	−9, −69, 24	−5.38	Both groups: EC increased	26
	Interaction effect	Bilateral midcingulate cortex	6, −24, 45	30.65	Estazolam: EC increased MSZRD: EC decreased	42
		L precentral gyrus	−18, −24, 69	21.04		32
ROI 6 (right dorsomedial parietooccipital sulcus of precuneus)						
*X‐to‐Y*	Interaction effect	Bilateral midcingulate cortex	3, −21, 48	27.26	Estazolam: EC decreased MSZRD: EC increased	41
*Y‐to‐X*	Group effect	R calcarine cortex	12, −66, 21	4.23	Post‐treatment: Estazolam < MSZRD	33
	Interaction effect	Bilateral midcingulate cortex	3, −21, 48	29.86	Estazolam: EC increased MSZRD: EC decreased	33
ROI 7 (left ventral precuneus, area 31)						
*Y‐to‐X*	Group effect	L Cerebellum Crus II	−36, −78, −42	−4.52	Both baseline and post‐treatment: Estazolam < MSZRD	45
ROI 8 (right ventral precuneus, area 31)						
*Y‐to‐X*	Group effect	L Cerebellum Crus II	−36, −75, −39	−4.36	Both baseline and post‐treatment: Estazolam < MSZRD	36

Abbreviations: EC, effective connectivity; L, left; MNI, Montreal Neurological Institute; MSZRD, Modified Suanzaoren Decoction Group; R, right; ROI, regions of interest.

### 3.3 Potential Gene Profiles Correlated With the EC Alterations

Genes associated with EC alterations of different ROIs were identified (Figures S3 and S4). Due to the limited number of genes, only those negatively correlated with *Y‐to‐X* EC changes in ROI2 and ROI8 were subjected to enrichment analysis, highlighting pathways related to cellular metabolism, ion and molecule transmembrane transport, vesicle and synaptic functions, and lipid metabolism.

## 4 Discussion

This longitudinal study investigated the ECs of precuneus subregions in CID patients before and after 6 weeks of MSZRD or estazolam treatment. At baseline, CID patients exhibited increased EC from the precuneus to the PoCG/PrCG and LG compared to HCs. At the same time, decreased EC from PoCG/PrCG, cerebellum crus VIIB, and calcarine cortex to precuneus was observed. MSZRD and estazolam treatment induced a distinct influence on the ECs, including ECs from the bilateral MCC to precuneus, as well as from bilateral MCC, left PrCG, and right SMG to precuneus. Both treatment groups exhibited a significantly increased EC from the left calcarine cortex to the precuneus after treatment. These findings suggested that the two treatments might exhibit both shared and distinct therapeutic mechanisms. Genes related to baseline EC alterations and potential functional pathways were identified, highlighting genetic involvement.

### 4.1 Increased ECs From the Precuneus to the Sensory‐Motor Network and Visual Network at Baseline

At baseline, CID patients showed increased ECs from the dorsal–anterior precuneus to PoCG/PrCG, and from the dorsal–posterior precuneus to left LG compared to HCs. This might indicate increased information flow from the precuneus to regions involved in the sensory‐motor network (SMN) and visual network (VN). The dorsal–anterior part of the precuneus is involved in sensorimotor functions (Margulies et al. 2009). The PoCG and PrCG, components of SMN, play a crucial role in processing environmental stimuli (Fasiello et al. 2022). Previous studies had found increased FC within the SMN, suggesting somatic hyperarousal and heightened sensitivity to sensory stimulation in CID patients (Fasiello et al. 2022; Killgore et al. 2013). Due to this hyperarousal state, the SMN received more sensory information from the precuneus, indicating a “top‐down” impairment in sensory processes. Additionally, the precuneus had a positive regulatory effect on the left LG. The dorsal–posterior part of the precuneus is involved in visual functions (Margulies et al. 2009). The LG, part of the occipital cortex, is primarily associated with visual signal processing and memory encoding. Zhou and colleagues have identified increased brain functional activity in the occipital cortex of CID patients, suggesting hyperarousal in visual functions (Zhou et al. 2017). The occipital cortex is responsible for interpreting visual stimuli, not only when perceiving real objects but also during visual‐mental imagery. Mental imagery can be triggered by environmental stimuli such as external noise or the ticking of a clock before sleep, potentially contributing to insomnia (Kim et al. 2019). Notably, mental imagery may occur even when the imagined stimuli differ from actual stimuli or in the absence of any physical stimuli (Clark and Mackay 2015; Dijkstra et al. 2021). Increased information flow from the precuneus to the occipital cortex in CID might reflect heightened sensitivity to environmental stimuli. These might lead to an increased generation of mental imagery and potentially result in difficulty initiating sleep.

### 4.2 Decreased ECs From the Sensory‐Motor Network and Visual Network to the Precuneus at Baseline

In CID patients, more information flows from the bilateral PoCG/PrCG, right calcarine cortex, and right cerebellum crus VIIB to precuneus was observed compared to HCs. The calcarine cortex, also known as the primary visual cortex, is located in the occipital lobe. Based on the above findings, a bidirectional influence between the precuneus and regions in the SMN and VN was observed. Specifically, when the precuneus sends positive regulatory signals to regions in the SMN and VN, these regions, in turn, exert deactivating effects on the precuneus. This might indicate the existence of a negative feedback loop in CID, potentially serving as a compensatory response to inhibit the abnormal hyperarousal state in CID. Moreover, positive correlations between the ECs from the right SMG to precuneus and ISI, PSQI, and HAMD scores were identified. The SMG is involved in sensory processing, similar to the PrCG, and plays a crucial role in integrating tactile information. Thus, these correlations might indicate that patients with more severe symptoms might exhibit weaker compensatory responses to their hyperarousal states. The right cerebellum crus VIIB has been proven to be engaged in cognitive and language‐demanding tasks, such as working memory and verb generation (Stoodley et al. 2012). Previous studies identified a higher nodal degree in cerebellar vermal VIIb in insomnia patients compared to HCs, suggesting a compensatory response to the cognitive impairment of insomnia (Ma et al. 2023). Thus, we speculated that the decreased EC from precuneus to cerebellum crus VIIB might reflect cognitive impairment in CID (Yang et al. 2023).

### 4.3 Comparison of ECs Between the Two Treatment Groups at Baseline

Compared to the estazolam group, the patients in the MSZRD group showed decreased ECs from the left cerebellum crus II to precuneus. A possible interpretation might be that CID patients had different medication histories (e.g., eszopiclone, lorazepam, and melatonin), which could impact these functional differences between patient groups. Although CID patients underwent a 2‐week washout period after enrollment, the effects of medications could not be completely ruled out. Moreover, ΔEC (EC changes from post‐ and pretreatment) between the MSZRD and estazolam groups were comparable, suggesting the baseline EC imbalance had a minimal effect on the longitudinal group‐by‐time interaction findings.

### 4.4 Abnormal EC Alterations of the Precuneus After 6 Weeks of Treatment

After 6 weeks of treatment, there were significant interaction effects on ECs between precuneus subregions and several regions, including MCC, SMG, and PrCG, suggesting MSZRD and estazolam might target similar pathways in the DMN, limbic network (LN), and SMN. The MCC, as part of the LN, functions in decision‐making, motor control, multisensory integration, and emotional regulation (Vogt 2016). Previous studies had reported limbic gray matter hypertrophy in insomnia patients, suggesting a functional hyperarousal in emotional processes (Yu et al. 2020). The SMG is involved in tactile sensory interpretation, spatial perception, limb localization, and emotional recognition. Kweon et al. reported a decreased amygdala‐SMG FC in CID patients, suggesting impaired “top‐down” regulation of the amygdala (Kweon et al. 2023). These impairments observed in LN and SMN suggested emotional and motor‐sensory dysfunction in CID patients. Both MSZRD and estazolam targeted these regions to modulate emotional and motor‐sensory functions in CID.

Although MSZRD and estazolam might target similar regions, they exerted distinct impacts. Specifically, increased ECs from regions in the LN and SMN to precuneus were observed after estazolam treatment, whereas decreased ECs from the same regions to precuneus after MSZRD treatment were identified. After MSZRD treatment, these regions had negative regulatory effects on the precuneus to alleviate the hyperarousal state in CID. Given the prevalent overactivity of the precuneus in insomnia, MSZRD might help the brain recover to a normal level by deactivating the precuneus. After estazolam treatment, these LN and SMN regions appeared to activate the precuneus. A possible explanation was that estazolam modulates GABAergic activity, providing a rapid sedative effect and immediate symptom relief that inhibits multiregional functions (Edinoff et al. 2021). The increased ECs might serve as a compensatory response, where the brain exerts positive regulatory effects to counterbalance the sedative impact. As supportive evidence, Feng et al. reported that insomnia patients after 4 weeks of estazolam treatment (1 mg/day) showed increased FC between the right hippocampus and left rostral anterior cingulate cortex/medial prefrontal cortex compared to HCs (Feng et al. 2019). The estazolam, while providing sedation, might heighten the activity of the precuneus as a form of adaptive regulation. These distinct altered EC patterns suggested different neural mechanisms of TCMs and BDZs in the treatment of insomnia. TCMs alleviate the hyperarousal state in CID while BDZs induce compensatory functional changes due to their rapid sedative effects. These findings also highlighted the potential of TCMs as viable alternatives to BDZs in the treatment of insomnia. The compensatory functional changes following BDZ treatment might be similar to the sensitization of excitatory glutamate receptors after prolonged GABA inhibition. These neuroadaptive processes account for tolerance, as many chronic users require higher dosages to attain the same effects (Edinoff et al. 2021). In contrast, TCMs seemed to provide a more balanced approach to regulating brain activity with a lower risk of dependence (Yeung et al. 2012). This suggests that TCMs like MSZRD could offer a safer, more sustainable alternative to BDZs, with potentially better treatment adherence due to fewer adverse effects and lower risk of dependence. Better adherence may improve treatment outcomes and aid in the overall recovery process for patients.

### 4.5 Potential Genetic Mechanisms Underlying EC Alterations

The genetic heritability of insomnia is approximately 38%–59% (Lind et al. 2015). However, genome‐wide analysis of insomnia only identified a small number of genetic loci (Jansen et al. 2019). Using neuroimaging–transcriptomics spatial analysis, we linked the neuroimaging data to the gene expression patterns in AHBA, uncovering genes associated with EC alterations in CID. Several genes were shared across various ROIs, indicating genetic involvement in CID. For instance, the expression level of *CDC5L* was correlated with EC alterations of both ROI1 and ROI8, while the expression level of *SESN2* was correlated with EC alterations of both ROI1 and ROI2. *SESN2* encodes sestrin‐2 protein, a stress‐inducible antioxidant protein that may help prevent neuroinflammation (He et al. 2020). Previous studies reported that insomnia patients might exhibit a reduced ability to resist oxidative stress (Gulec et al. 2012). Su et al. supported this notion, revealing lower levels of sestrin‐2 in CID patients compared to HCs (Su et al. 2024). In conjunction with our findings, *SESN2* expression might play a role in the neurobiology of CID. Moreover, some EC‐related genes were enriched in pathways related to synaptic functions. This suggests that the observed EC disruptions in the SMN and VN, which may reflect the hyperarousal state in CID, could be linked to these biological processes. In fact, hyperarousal before sleep might lead to restless REM sleep, which in turn disrupts synaptic plasticity (Riemann et al. 2010). For instance, a study observed increased expression of *DNM1* in *BTBD9* (the top gene associated with insomnia) mutant mice (DeAndrade et al. 2012), which encodes the synaptic protein dynamin (Van Someren 2021) and mediates the sleep‐disruptive effects of presleep arousal (Suzuki et al. 2013). Thus, the changes of synaptic plasticity might provide insights into the potential molecular mechanisms underlying CID.

### 4.6 Limitations

However, several limitations should be acknowledged. First, this study exclusively recruited patients diagnosed with Yin‐deficiency and fire‐excess syndrome, a condition specifically targeted by MSZRD. While this design ensured the appropriateness of the intervention, it may limit the generalizability of our findings to the broader population of CID patients. Future studies should consider including participants with diverse TCM syndromes or nonsyndrome‐based CID diagnoses to enhance external validity. Second, treatment allocation was based on patient preference rather than randomization, which may introduce selection bias. Treatment preference may be related to expectation effects or symptom characteristics, potentially influencing treatment response and confounding the observed group‐by‐time effects. Future studies using randomized, double‐blind designs are needed to validate these findings. Third, although CID patients underwent a 2‐week washout period after enrollment, the effects of prior medications could not be entirely excluded. Future studies should control for medication use by recruiting drug‐naïve patients to better clarify potential pharmacological influences. Finally, in the neuroimaging–transcriptomic analysis, the fMRI data were derived from CID patients, whereas the gene expression data were obtained from the AHBA database. The AHBA dataset includes a limited number of donors with an imbalanced sex distribution, and the gene expression maps represent population‐level normative data rather than patient‐specific measurements. Consequently, the results may be influenced by demographic and biological differences (e.g., age, sex, and ancestry) between the imaging cohort and the transcriptomic dataset. Therefore, these findings should be interpreted cautiously and considered exploratory.

## 5 Conclusions

Our findings suggested the crucial role of the precuneus in the neuropathological and treatment mechanisms of CID at a subtler level. We observed bidirectional interactions between the precuneus subregions and regions in the SMN and VN in CID, which may reflect both hyperarousal states and compensatory responses aimed at mitigating abnormal hyperarousal in these patients. TCMs and BDZs might target the SMN and LN regions, but exert distinct impacts. TCM treatment tended to alleviate the hyperarousal state in CID, while BDZs induced a positive regulation of the precuneus as a neuroadaptive process due to their rapid sedative effects. These findings highlighted the potential of TCMs as viable alternatives to BDZs in the treatment of insomnia, as TCMs provide a more balanced approach to regulating brain activity. Using neuroimaging–transcriptomics spatial analysis, we identified genes related to these ECs and potential pathophysiological pathways.

## Author Contributions

**Zhiguo Guo**: writing – original draft, writing – review and editing, formal analysis, data curation. **Lixia Chen**: conceptualization, methodology, writing – review and editing, funding acquisition. **Ping Yao**: conceptualization, data curation. **Dongsheng Lv**: conceptualization, data curation. **Yonggui Yuan**: conceptualization, data curation. **Leyi Zhang**: data curation, writing – original draft, writing – review and editing, formal analysis. **Haohao Yan**: conceptualization, data curation. **Yiding Han**: data curation, writing – review and editing, formal analysis, writing – original draft. **Wenbin Guo**: writing – review and editing, conceptualization, methodology, data curation, funding acquisition. **Jingping Zhao**: data curation, conceptualization.

## Funding

This study was supported by the National Natural Science Foundation of China (Grant No. 82171508).

## Ethics Statement

The study was approved by the Medical Ethics Committee of Inner Mongolia Autonomous Region Mental Health Center.

## Consent

All the participants gave written informed consent. All the authors have consented to publish this manuscript.

## Conflicts of Interest

The authors declare no conflicts of interest.

## Supporting information




**Supporting Information**: brb371448‐sup‐0001‐SuppMat.docx

## Data Availability

Data are available upon reasonable request to the corresponding author.
