# Supplementary material for: Altered Effective Connectivity of Precuneus Subregions and Associated Gene Profiles in Chronic Insomnia Disorder Patients Before and After Treatment
Source: Brain Behav. 2026 May 19;16(5):e71448. doi: 10.1002/brb3.71448 (PMC13580868; doi:10.1002/brb3.71448)
Supplement: Supplementary file 1 [file BRB3-16-e71448-s001.docx]

**Supplementary materials**

### **Altered effective connectivity of precuneus subregions and associated gene profiles in chronic insomnia patients before and after treatment**

**Authors:**

Zhiguo Guo^1, #^, Leyi Zhang^2, #^, Yiding Han^2, #^, Haohao Yan^2^, Ping Yao^1^, Dongsheng Lv ^1^, Yonggui Yuan^3^, Jingping Zhao^2^, Lixia Chen ^1, *^, and Wenbin Guo^2, *^

**Affiliation/address:**

^1^ Inner Mongolia Autonomous Region Mental Health Center, Hohhot 010010, Inner Mongolia Autonomous Region, China.

^2^ Department of Psychiatry, National Clinical Research Center for Mental Disorders, and National Center for Mental Disorders, The Second Xiangya Hospital of Central South University, Changsha 410011, Hunan, China.

^3^ Department of Psychosomatics and Psychiatry, Zhongda Hospital, School of Medicine, Jiangsu Provincial Key Laboratory of Brain Science and Medicine, Southeast University, Nanjing, 210009, China.

\# These authors contributed equally to this work.

Corresponding authors:

Wenbin Guo

Department of Psychiatry, National Clinical Research Center for Mental Disorders, and National Center for Mental Disorders, The Second Xiangya Hospital of Central South University, Changsha 410011, Hunan, China.

E-mail: guowenbin76@csu.edu.cn

Tel.: +86 731 85360921

Lixia Chen

Inner Mongolia Autonomous Region Mental Health Center, Hohhot 010010, Inner Mongolia Autonomous Region, China.

E-mail: clx001@sohu.com

**Methods**

**Participants**

The diagnostic process for the "yin deficiency and fire excess" syndrome type is conducted in accordance with the symptom characteristic of this syndrome. The comprehensive evaluation through the four traditional diagnostic methods of TCM: inspection, auscultation, inquiry, and palpation. It is tailored to identify and confirm symptoms such as headaches, irritability, night sweats, dry mouth, palpitations, a red tongue, and a rapid pulse, which are indicative of "yin deficiency and fire excess."

1) Inspection: Patients are observed for facial redness, especially in the afternoon or evening, and for a red tongue with little or no coating, which may appear dry and thin. The overall physical appearance, including a lean physique and a haggard look, is also noted.

2) Auscultation: The patient’s voice quality is assessed for hoarseness or a low pitch, and their breathing rate is observed, particularly noting any rapid breathing during emotional arousal. Any unusual body odors, such as dry mouth or bad breath, are also recorded.

3) Inquiry: A detailed inquiry is conducted regarding the patient’s sleep patterns, including difficulty falling asleep, frequent awakenings, and early morning awakenings. Accompanying symptoms such as five-center heat (palms, soles, and chest), night sweats, dry mouth and throat, dizziness, tinnitus, soreness in the lower back and knees, palpitations, forgetfulness, and emotional irritability are also explored. Additionally, lifestyle habits, dietary patterns, and emotional stress levels are assessed.

4) Palpation: The pulse is felt for a thin and rapid quality, indicating a fine and wiry pulse. The skin is checked for dryness and warmth, and the abdomen is gently palpated to check for any bloating or tenderness.

**Granger causality analysis**

In this study, time series of bilateral precuneus were defined as seed time series x, and time series y denoted all voxels in the brain. GCA calculates the causal effect of the ROI on the other regions in the whole brain (X to Y effect) and Y to X effect. An increased EC from X to Y indicates that activity in region X exerts a positive influence on the activity in region Y. Similarly, a decreased EC from X to Y suggests that the activity of region X exerts a negative influence on the activity of region Y. These GCA maps were then generated through Fisher z-transformation. Notably, a primary concern of GCA involves the potential confounding effects of the time lag between hemodynamic response function and fMRI data. The inhibitory or excitatory properties of neuronal clusters and the size of neuronal cluster activation might reduce the accuracy of causality identification across the brain.

Figure S1. The changes in clinical assessments over time in the two patient groups were measured using a mixed effects model. PSQI = Pittsburgh Sleep Quality Index; PSG = polysomnography.

Figure S2. Correlations between EC alterations of ROIs and clinical characteristics. ROI = Regions of interest; EC = effective connectivity; L=left; R = right; PSQI = Pittsburgh Sleep Quality Index; ISI = Insomnia Severity Index; HAMD = Hamilton Depression Rating Scale; ROI 1 = dorsal-central precuneus, medial area 7 (L); ROI 2 = dorsal-central precuneus, medial area 7 (R); ROI 3 = dorsal-anterior precuneus, medial area 5 (L); ROI 4 = dorsal-anterior precuneus, medial area 5 (R); ROI 5 = dorsal-posterior precuneus, dorsomedial parietooccipital sulcus (L); ROI 6 = dorsal-posterior precuneus, dorsomedial parietooccipital sulcus (R); ROI 7 = ventral precuneus, area 31 (L); ROI 8 = ventral precuneus, area 31 (R).

Figure S3. The correlation heatmap illustrates the correlation coefficients of genes associated with EC coefficient alterations based on ROIs. Gene enrichment analysis was performed separately for positively and negatively correlated genes, and the results are displayed in the bubble diagram. EC = effective connectivity; ROI = regional of interest; ROI 1 = dorsal-central precuneus, medial area 7 (L); ROI 2 = dorsal-central precuneus, medial area 7 (R); ROI 4 = dorsal-anterior precuneus, medial area 5 (R); ROI 5 = dorsal-posterior precuneus, dorsomedial parietooccipital sulcus (L).

Figure S4. The correlation heatmap illustrates the correlation coefficients of genes associated with EC coefficient alterations based on ROIs. Gene enrichment analysis was performed separately for positively and negatively correlated genes, and the results are displayed in the bubble diagram. EC = effective connectivity; ROI = regional of interest; ROI 6 = dorsal-posterior precuneus, dorsomedial parietooccipital sulcus (R); ROI 7 = ventral precuneus, area 31 (L); ROI 8 = ventral precuneus, area 31 (R).

Table S1. Coordinates of subdivisions of the precuneus in the Brainetome Atlas.

| Anatomical and modified Cyto-architectonic descriptions | MNI (X, Y, Z) | |
| --- | --- | --- |
|  | Left | Right |
| dorsal-central precuneus, medial area 7 | -5, -63, 51 | 6, -65, 51 |
| dorsal-anterior precuneus, medial area 5 | -8, -47, 57 | 7, -47, 58 |
| dorsal-posterior precuneus, dorsal parietooccipital sulcus | -12, -67, 25 | 16, -64, 25 |
| ventral precuneus, area 31 | -6, -55, 34 | 6, -54, 35 |

MNI = Montreal Neurological Institute.

Table S2. Demographic and clinical characteristics.

| Variables | Suanzaoren decoction group (n=52) | Estazolam group (n=30) | Healthy controls (n=80) | *p*-value |
| --- | --- | --- | --- | --- |
| Age (years) | 45.79±11.86 | 46.57±10.41 | 41.87±10.30 | 0.956 ^a^ |
| Gender (male/female) | 13/39 | 3/27 | 18/62 | 0.247 ^b^ |
| Educational level (years) | 14.04±2.91 | 13.93±2.88 | 14.19±3.31 | 0.871 ^a^ |
| PSQI | | | | |
| Subjective sleep quality | 2.47±0.54 | 2.59±0.57 | - | 0.307^c^ |
| Sleep latency | 2.69±0.84 | 2.79±0.49 | - | 0.439^c^ |
| Sleep duration | 2.49±0.70 | 2.48±0.87 | - | 0.735^c^ |
| Sleep disturbances | 2.49±0.83 | 2.69±0.71 | - | 0.227^c^ |
| Use of sleep mediation | 1.47±0.50 | 1.34±0.55 | - | 0.213^c^ |
| Daytime dysfunction | 1.31±1.24 | 1.83±1.17 | - | 0.077^c^ |
| Total score | 14.35±3.02 | 15.53±2.86 | - | 0.085^d^ |
| ISI | 4.98±3.33 | 5.00±3.79 | - | 0.969^c^ |
| PSG | | | | |
| Sleep efficiency, % | 78.63±13.51 | 80.60±11.88 | - | 0.547^c^ |
| Sleep latency (min) | 21.13±16.94 | 22.68±17.85 | - | 0.583^c^ |
| REM latency (min) | 102.35±151.04 | 107.17±63.02 | - | 0.063^c^ |
| REM sleep (min) | 76.85±30.99 | 86.95±29.50 | - | 0.199^c^ |
| N1 (min) | 58.71±30.55 | 65.03±34.39 | - | 0.519^c^ |
| N2 (min) | 187.29±54.71 | 193.42±46.23 | - | 0.607^d^ |
| N3 (min) | 23.82±24.04 | 20.10±18.87 | - | 0.458^c^ |
| Total sleep time (min) | 356.50±69.49 | 365.95±68.16 | - | 0.552^d^ |
| HAMA | 20.79±5.81 | 23.13±4.93 | - | 0.066^c^ |
| HAMD | 10.15±4.04 | 11.00±4.46 | - | 0.279^c^ |

Notes: Means and standard deviations are reported for all demography and clinical characteristics. PSQI = Pittsburgh Sleep Quality Index; ISI = Insomnia Severity Index; PSG = polysomnography; HAMA = Hamilton anxiety scale; HAMD = Hamilton’s Depression Scale.

^a^ The *p*-values were obtained by Kruskal-Wallis tests.

^b^ The *p*-value for sex distribution was obtained by a Chi-Square tests.

^c^ The *p*-values were obtained by Mann-Whitney U tests.

^d^ The *p*-values were obtained by Two sample T tests.

Table S3. The clinical variables of patients before and after treatment.

| Variables | Suanzaoren decoction group (N = 41) | |  | | Estazolam group (N = 25) | |
| --- | --- | --- | --- | --- | --- | --- |
|  | Baseline | Post-treatment |  |  | Baseline | Post-treatment |
| PSQI | | |  |  | |  |
| Subjective sleep quality | 2.45±0.55 | 1.05±0.45 |  | 2.60±0.58 | | 1.08±0.49 |
| Sleep latency | 2.75±0.84 | 1.57±0.81 |  | 2.76±0.52 | | 2.00±0.76 |
| Sleep duration | 2.40±0.74 | 1.25±0.93 |  | 2.68±0.56 | | 1.56±0.96 |
| Sleep efficiency | 2.47±0.82 | 1.35±1.10 |  | 2.80±0.50 | | 1.56±1.26 |
| Sleep disturbances | 1.48±0.51 | 1.15±0.43 |  | 1.40±0.58 | | 1.00±0.29 |
| Use of sleep mediation | 1.53±1.24 | 1.80±1.29 |  | 1.84±1.21 | | 2.28±1.02 |
| Daytime dysfunction | 1.25±1.03 | 0.57±0.68 |  | 1.52±0.87 | | 0.92±0.76 |
| Total score | 14.44±3.19 | 8.80±3.05 |  | 15.68±2.32 | | 10.40±2.55 |
| ISI | 20.71±5.69 | 10.02±4.84 |  | 23.20±4.93 | | 10.28±4.22 |
| PSG | | |  |  | |  |
| Sleep efficiency, % | 77.68±15.20 | 83.63±10.78 |  | 80.63±12.25 | | 81.52±11.86 |
| Sleep latency, min | 20.51±16.71 | 19.98±26.84 |  | 21.78±18.99 | | 28.74±30.66 |
| REM latency, min | 84.20±48.24 | 97.10±45.53 |  | 102.78±66.99 | | 89.20±45.18 |
| REM sleep, min | 77.29±32.71 | 86.34±34.37 |  | 84.18±28.18 | | 90.44±31.65 |
| N1, min | 55.00±31.22 | 52.59±29.33 |  | 60.48±30.01 | | 52.00±28.58 |
| N2, min | 186.63±53.47 | 195.68±49.33 |  | 190.98±51.13 | | 192.06±55.87 |
| N3, min | 23.59±25.19 | 37.44±33.70 |  | 17.96±16.61 | | 35.56±36.93 |
| Total sleep time, min | 355.44±69.02 | 368.59±56.31 |  | 354.56±66.57 | | 374.84±66.36 |

We provided the mean and standard deviation of the scale scores. PSQI = Pittsburgh Sleep Quality Index; ISI = Insomnia Severity Index; PSG = polysomnography.

Table S4. The comparison of clinical variables before and after treatment.

| Variables | Time | |  | Group | |  | Time×Group | |
| --- | --- | --- | --- | --- | --- | --- | --- | --- |
|  | Estimate (95% CI) | p-value |  | Estimate (95% CI) | p-value |  | Estimate (95% CI) | p-value |
| PSQI | | | | | | | | |
| Subjective sleep quality | 0.02 (-0.01, 0.05) | 0.110 |  | 0.68 (-0.31, 1.68) | 0.174 |  | -0.01 (-0.03, 0.01) | 0.269 |
| Sleep latency | 0.03 (-0.01, 0.06) | 0.149 |  | 0.24 (-1.02, 1.50) | 0.707 |  | -0.01 (-0.04, 0.01) | 0.307 |
| Sleep duration | 0.07 (0.04, 0.10) | <0.001 |  | 2.26 (1.09, 3.43) | <0.001 |  | -0.05 (-0.07, -0.02) | <0.001 |
| Sleep efficiency | 0.07 (0.02, 0.12) | 0.006 |  | 2.57 (0.81, 4.33) | 0.005 |  | -0.05 (-0.08, -0.02) | 0.004 |
| Sleep disturbances | 0.01 (-0.02, 0.03) | 0.607 |  | 0.25 (-0.54, 1.05) | 0.531 |  | >-0.01 (-0.01, 0.01) | 0.670 |
| Use of sleep mediation | 0.03 (-0.03, 0.09) | 0.419 |  | -0.06 (-2.35, 2.23) | 0.957 |  | <0.01 (-0.04, 0.04) | 0.987 |
| Daytime dysfunction | 0.02 (-0.02, 0.06) | 0.301 |  | 0.59 (-0.96, 2.14) | 0.450 |  | -0.01 (-0.04, 0.02) | 0.40 |
| Total score | 0.30 (0.18, 0.42) | <0.001 |  | 7.81 (3.82, 11.82) | <0.001 |  | -0.16 (-0.24, -0.09) | <0.001 |
| ISI | 0.21 (-0.01, 0.43) | 0.057 |  | 8.08 (0.50, 15.66) | 0.037 |  | -0.12 (-0.27, 0.32) | 0.120 |
| PSG | | |  |  | |  |  | |
| Sleep efficiency (%) | -0.32 (-1.00, 0.36) | 0.349 |  | -7.42 (-30.88, 16.05) | 0.530 |  | 0.26 (-0.20, 0.72) | 0.260 |
| Sleep latency (min) | 0.48 (-0.83, 1.78) | 0.469 |  | 6.17 (-38.67, 51.00) | 0.784 |  | -0.28 (-1.16, 0.61) | 0.534 |
| REM latency (min) | 0.15 (-2.85, 3.14) | 0.923 |  | 13.82 (-89.23, 116.87) | 0.790 |  | 0.31 (-1.72, 2.33) | 0.764 |
| REM sleep (min) | 0.75 (-0.82, 2.32) | 0.342 |  | 31.23 (-22.71, 85.17) | 0.243 |  | -0.60 (-1.66, 0.46) | 0.265 |
| N1 (min) | -0.99 (-2.20, 0.22) | 0.108 |  | -34.80 (-76.50, 6.90) | 0.100 |  | 0.87 (0.05, 1.69) | 0.039 |
| N2 (min) | -0.96 (-3.54, 1.61) | 0.457 |  | -37.70 (-126.25, 50.85) | 0.398 |  | 0.98 (-0.76, 2.72) | 0.265 |
| N3 (min) | 1.24 (-0.20, 2.68) | 0.090 |  | 50.45 (1.05, 99.86) | 0.045 |  | -1.16 (-2.13, -0.18) | 0.020 |
| Total sleep time (min) | 0.49 (-3.01, 3.40) | 0.779 |  | 3.43 (-116.98, 123.85) | 0.955 |  | -0.20 (-2.57, 2.16) | 0.864 |

CI = Confidence Intervals; PSQI = Pittsburgh Sleep Quality Index; ISI = Insomnia Severity Index, PSG = polysomnography.

Table S5. The baseline difference between two patient groups.

| ROIs | Brain regions | MNI  (x, y, z) | T value | Cluster size |
| --- | --- | --- | --- | --- |
| ROI 7 (left ventral precuneus, area 31 of precuneus) | | | | |
| *Y-to-X* | L Cerebellum Crus II | -33 -75 -42 | -4.2 | 31 |
| ROI 8 (right ventral precuneus, area 31 of precuneus) | | | | |
| *Y-to-X* | L Cerebellum Crus II | -30 -75 -42 | -4.17 | 22 |

ROI = Regions of interest; MNI = Montreal Neurological Institute.

Table S6. The correlations between the effective connectivity of ROIs and clinical characteristics.

| ROIs | Corelated clinical characteristics | Brain Regions | MNI  (x, y, z) | R values | Correlations | Cluster size |
| --- | --- | --- | --- | --- | --- | --- |
|  | ROI1 (left dorsal-central precuneus, medial area 7) | | | | | |
| *Y-to-X* | ISI | R Supramarginal/Postcentral Gyrus | 69 -15 24 | 0.45 | Positive | 48 |
|  | ROI2 (right dorsal-central precuneus, medial area 7) | | | | | |
| *X-to-Y* | PSQI | L Cerebellum Crus II | -24 -78 -45 | 0.46 | Positive | 35 |
| *Y-to-X* | PSQI | R Supramarginal Gyrus | 48 -42 21 | 0.48 | Positive | 53 |
|  | ROI3 (left dorsal-anterior precuneus, medial area 5) | | | | | |
| *Y-to-X* | ISI | L Superior Temporal Gyrus | -63 -39 21 | 0.49 | Positive | 45 |
|  |  | R Supramarginal Gyrus | 51 -39 18 | 0.44 | Positive | 63 |
|  | ROI4 (right dorsal-anterior precuneus, medial area 5) | | | | | |
| *X-to-Y* | PSQI | L Superior Medial Frontal Gyrus | -6 45 36 | -0.47 | Negative | 47 |
| *Y-to-X* | ISI | R Superior Temporal Gyrus | 60 -18 3 | 0.47 | Positive | 63 |
|  |  | L Superior Temporal Gyrus | -66 -39 18 | 0.49 | Positive | 58 |
|  | ROI5 (left dorsomedial parietooccipital sulcus of precuneus) | | | | | |
| *Y-to-X* | HAMD | L Calcarine Gyrus/Precuneus | -21 -51 6 | -0.44 | Negative | 31 |
|  | ROI7 (left ventral precuneus, area 31) | | | | | |
| *Y-to-X* | HAMD | L Superior Frontal Gyrus | -18 42 39 | -0.59 | Negative | 46 |
|  | ROI8 (right ventral precuneus, area 31) | | | | | |
| *Y-to-X* | HAMD | R Supramarginal Gyrus | 63 -24 33 | 0.46 | Positive | 50 |
|  |  | L Superior Frontal Gyrus | -18 42 39 | -0.50 | Negative | 39 |

ROI = Regions of interest; MNI = Montreal Neurological Institute; L=left; R = right; PSQI = Pittsburgh Sleep Quality Index; ISI = Insomnia Severity Index; HAMD = Hamilton Depression Rating Scale.

Table S7. Results of interaction effects between demographic/clinical factors and treatment-related changes in functional connectivity.

| Effects | Estimate | Standard Error | T values | P |
| --- | --- | --- | --- | --- |
| Sex | -1.85 | 3.49 | -0.53 | 0.59 |
| Sex×Group | -4.03 | 12.52 | -0.32 | 0.74 |
| Sex×Time | 0.048 | 0.06 | 0.72 | 0.46 |
| Age | -0.39 | 2.57 | -0.15 | 0.88 |
| Age×Group | -2.46 | 4.05 | -0.60 | 0.54 |
| Age×Time | 0.033 | 0.048 | 0.68 | 0.49 |
| ISI | 3.13 | 3.04 | 1.02 | 0.30 |
| ISI×Group | 0.11 | 8.41 | 0.01 | 0.98 |
| ISI×Time | -0.04 | 0.06 | -0.64 | 0.52 |

ISI = Insomnia Severity Index.
